# Systematic analysis of non-structural protein features for the prediction of PTM function potential by artificial neural networks

**DOI:** 10.1371/journal.pone.0172572

**Published:** 2017-02-22

**Authors:** Henry M. Dewhurst, Matthew P. Torres

**Affiliations:** School of Biological Sciences, Georgia Institute of Technology, Atlanta, Georgia, United States of America; Harbin Institute of Technology Shenzhen Graduate School, CHINA

## Abstract

Post-translational modifications (PTMs) provide an extensible framework for regulation of protein behavior beyond the diversity represented within the genome alone. While the rate of identification of PTMs has rapidly increased in recent years, our knowledge of PTM functionality encompasses less than 5% of this data. We previously developed SAPH-ire (Structural Analysis of PTM Hotspots) for the prioritization of eukaryotic PTMs based on function potential of discrete modified alignment positions (MAPs) in a set of 8 protein families. A proteome-wide expansion of the dataset to all families of PTM-bearing, eukaryotic proteins with a representational crystal structure and the application of artificial neural network (ANN) models demonstrated the broader applicability of this approach. Although structural features of proteins have been repeatedly demonstrated to be predictive of PTM functionality, the availability of adequately resolved 3D structures in the Protein Data Bank (PDB) limits the scope of these methods. In order to bridge this gap and capture the larger set of PTM-bearing proteins without an available, homologous structure, we explored all available MAP features as ANN inputs to identify predictive models that do not rely on 3D protein structural data. This systematic, algorithmic approach explores 8 available input features in exhaustive combinations (247 models; size 2–8). To control for potential bias in random sampling for holdback in training sets, we iterated each model across 100 randomized, sample training and testing sets—yielding 24,700 individual ANNs. The size of the analyzed dataset and iterative generation of ANNs represents the largest and most thorough investigation of predictive models for PTM functionality to date. Comparison of input layer combinations allows us to quantify ANN performance with a high degree of confidence and subsequently select a top-ranked, robust fit model which highlights 3,687 MAPs, including 10,933 PTMs with a high probability of biological impact but without a currently known functional role.

## Introduction

As improved techniques in mass spectrometry have quickened the identification of PTMs, the rate of experimental observation of these modifications has far outpaced our ability to qualify their function. Furthermore, it has been demonstrated that biological function is neither necessary nor equivalent amongst the observed PTMs [3–5]. Given the resource commitment required for confirmation of function for each PTM, methods for prioritizing modifications with currently unknown function based on predicted probability of function are required.

Ideally, predictive models would directly diagnose the functional significance of a PTM site. However, this is unfeasible without an initial framework that establishes a relationship between biological features of PTM sites, or rather Modified Alignment Positions (MAPs) (e.g. sequence conservation, protein interface residence, observation frequency, neighbors, etc.) and PTM function status (functional or non functional). While PTM site features can be precisely defined, this is rarely true for PTM functional status, which is a product of diverse experimental paradigms and procedures that evolve through historical time. Moreover, failed attempts to determine PTM functionality are neither conclusive nor commonly reported. Consequently, the classification problem becomes one of discerning PTMs with reported evidence (or high suspicion) of function from those for which a functional role for the modification has not yet been reported. Prediction of PTM function potential then becomes a classification problem whereby each PTM is ranked by the combination of its MAP features and its similarity (or difference) from MAP feature combinations that have been linked with biological function *a priori*.

Artificial Neural Networks (ANNs) provide a systematic process whereby quantifiable input features are scaled and integrated through an iterative training process intended to maximize classification accuracy based on a training set of pre-classified data [6]. We have previously validated a combination of MAP features that, when integrated through ANN models, enable the effective prediction of MAPs where positive functional status is known *a priori* (i.e. known-functional MAPs; KF-MAPs) [2]. Consequently, MAPs with unknown functional significance (UF-MAPs), which have feature combinations similar to those of KF-MAPs, are scored accordingly and rank ordered based on a predicted probability of known function [P(Known = 1)]. Used in this way, ANN models significantly outperform single-feature logistic regression models for the identification of KF-MAPs that are not included in the training set [2]. In addition, they minimize the dominance of any one feature alone (e.g. PTM observation frequency)–enabling comparisons between a diverse range of MAPs within or across protein family [2]. The resulting outcome from the ANN model is a prioritized set of MAPs (and therefore PTM sites) that have significant potential for biological function.

PTMs, in their ability to impact protein behavior through (in)activation, allosteric regulation, and modification of protein binding sites, exert their regulatory influence through alterations that are fundamentally structural [7]. Accordingly, structural features have often been included in the confirmation of PTM localization, crosstalk between modifications, and investigation into biological functions [8–12]. Where structural data is available, features derived from such data have been shown to correlate with probability of biological function [12,13]. Moreover, we have shown previously that structural features such as solvent accessible surface area (SASA) of the modified residue, contribute significantly to ANN models in which they are included as an input feature [2]. However, less than one quarter of experimentally verified eukaryotic PTMs can be associated with sufficient structural data from their carrier protein or other members within a protein family. Given the current relative rates of expansion of publically available data, the gap between quantity of experimentally observed PTMs and adequately resolved crystal structures will continue to widen. Thus, models that can accurately classify KF-MAPs without the use of structural data input features would effectively increase the analytical coverage to the growing landscape of structurally unresolved (“structure-free”) proteins with experimentally verified PTMs.

We set out here to systematically examine all possible combinations of structure-free MAP features as input variables for ANN models trained for PTM function potential classification. Using SAPH-ire coupled with an automated method implemented in R (SAPH-ire FPx), we have tested 24,700 ANN models trained to rank order the function potential of discrete MAPs. As a result, we have generated 2.5 million probabilities across 84,438 MAPs harboring 115,389 distinct PTMs—an expansion beyond the current structural dataset of more than 64,000 PTMs. Similar to our previous results, neural network models consistently outperform the use of any one single feature logistic regression model, including the source count for mass-spectrometry based reports that are generally considered predictive outright. Furthermore, we observe a maximum receiver operating characteristic (ROC) with an area under the curve (AUC) of 0.8085 and a robust fit for several top-ranking ANN models that include a variety of MAP feature combinations. The top model highlights 3,687 high-probability UF-MAPs—containing nearly 11,000 individual modifications—representing PTMs with a high potential for functional impact that represent possible regulatory elements for their respective protein families.

## Methods

### Assembly of structure-free dataset

The SAPH-ire method—described in detail in previous publications—is summarized here with alterations included for the structure-free dataset [2,13]. A generalized processing schematic for the application of SAPH-ire to the structure-free dataset is illustrated in (S1 Fig). An internal MySQL database was created to store a manually curated subset of the experimentally observed eukaryotic PTMs obtained from dbPTM3 [14]. Certain PTM types were excluded where the modification could not be reasonably mapped to a specific residue (e.g. cleavage events). Any PTMs that lacked a valid PubMed ID (PMID) for identification were also excluded. Each PTM was mapped to a UniProt identifier (UID) and any obsolete entries were removed [15]. The sequences of each PTM-bearing UID were clustered into families according to InterPro “family” and “superfamily” classification schema [16]. The multi-FASTA files generated for each family were subsequently aligned using the MUSCLE algorithm with default parameters [17]. The structure-free dataset was aggregated into 83,438 MAPs across 4,814 protein families containing 20,020 unique UIDs and contains 115k PTMs. Data concerning evidence of function for 3,998 PTMs contained in the dataset was obtained from PhosphoSitePlus [18].

Eight protein and familial features were calculated and included as available input layers in ANN models. (A) The PTM count (PTMc) was obtained by summing number of distinct PTM sites observed within a MAP. (B) The PTM residue conservation (PRC) was determined from the ratio of modifiable residues (by any of the aligned PTM types; e.g., coincidence of phosphorylation and ubiquitination yields the set of modifiable residues = {S,Y,T,K}) to the number of residues aligned at a MAP (excluding gaps). (C) The mean intrinsic disorder (DIS), as measured by the IUPred algorithm, across all residues aligned within the MAP [19]. (D) The count of unique taxonomic IDs (organism IDs; OIDc) for proteins contributing a PTM to a MAP. (E) The number of times a PTM has been independently observed (the MS source count; MSSrc). Three additional measures examine neighboring modifications in alignment space using a window of +/-2 alignment positions (gaps included)–an approach that has been established for accounting for MSA variability and the significance of PTM clusters [20]. (F) The count of neighboring MAPs (Nc) which ranges from 0 to a maximum of 4. (G) The count of neighboring PTMs (NPTMc) which sums the PTMc for MAPs within the window (excluding the PTMc for the reference MAP). (H) The count of neighboring MAPs containing PTMs of known function (KF-MAPs) which, like Nc, ranges from 0 to a maximum of 4.

Although not included in the input layer, the number of PMIDs associated with confirmation of biological function for a PTM (known function source count; KFSC) in the PhosphoSitePlus data were recorded. As this source count provides a measure of confidence in functional assignment and was not revealed in model training, it provides a valuable metric for ANN performance. Redundant PMID—PTM relations were removed to ensure the count of unique PTM function studies was incorporated.

### Neural network generation

ANNs were constructed iteratively in R using the neuralnet package [21]. Parallelization was achieved using the doParallel package [22]. In each case, neural network models were trained and validated using the subset of MAPs belonging to families with at least one known functional PTM site. Input datasets were queried within R from a local MySQL database containing the structure-free SAPH-ire output using the RMySQL package [23]. ANNs were created for every possible linear combination the 8 available input factors for models ranging from 2 to 8 factors—yielding a total of 247 unique input layers. Each input factor combination was applied across 100 randomly selected training and validation sets using a 33% holdback for training. Use of random seeds ensured consistency and reproducibility of randomized training and testing sets across different input factor combinations. In total, 24,700 individual ANN models were generated with unique input layer and sampling combinations.

### Measuring ANN performance

Model performance and fit was assessed using ROC curves and their associated measures (i.e., AUC) generated using the ROCR package with the application of each ANN to the entire dataset [24]. The ROC curve was selected as the primary performance metric as a generally understood and readily interpreted scale for measuring accuracy warrants use of the ROC for neural networks [25]. Comparisons between different input layer combinations were made by calculating the aggregate ROC AUC statistics across all 100 randomized sample sets. Using the top-model (based on mean ROC AUC), a conservative cutoff for identifying high P(Known = 1) UF-MAPs was employed wherein the upper 90% of P(Known = 1) values for KF-MAPs with KFSC of 5+ were considered function potential hotspots, and recommended for empirical validation of biological function.

## Results and discussion

### The “structure-free” dataset

**Fig 1:**
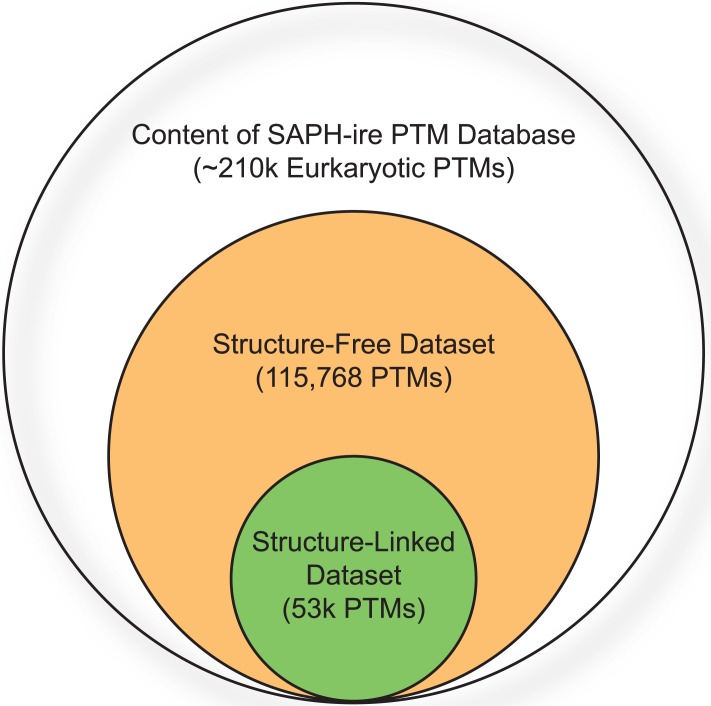
Relative quantities of structure-linked and structure-free PTM data analyzed by SAPH-ire. The Venn diagram illustrates the relative quantities of PTM data (individual modification sites) which are analyzable by traditional SAPH-ire and those included in the structure-free dataset as compared to the total number of experimentally verified PTMs curated within the internal MySQL database of eukaryotic modifications used in this study.

The SAPH-ire model has demonstrated exceptional predictive power for PTMs contained within the dataset of 3D structure-linked proteins. Specifically, the solvent accessible surface area (SASA), extracted from 3D structures and used as a feature in neural network models, ranks as the second best single predictive feature next to the count of family members with observed modifications within a MAP (referred to as PTM count) [2]. However, of the 213,022 experimentally observed eukaryotic PTMs contained within the comprehensive dataset available at the time of this study, only ~50,000 are associated with structure-linked features (directly or through familial relationship), which severely limits the quantity of PTMs available for analysis (Fig 1). Moving to a model that does not require structural input more than doubles the number of analyzable PTMs as compared to previous analyses, enabling function potential prediction for 115,389 PTMs across 4,814 protein families. Within this set, 63 different modification types are observed across 83,438 MAPs– 3,998 corresponding to KF-MAPs and 80,440 to UF-MAPs utilized for randomized ANN training and validations sets [18].

### Systematic analysis of input factors reveals several high-performance predictive models

Theoretically, artificial neural network models can be built from an infinite number of input factors. In practice, the number ranges from as few as 2 to as many as 160 billion [26]. Protein structure-based ANN models with as few as 6 input factors—including PTM count (PTMc), SASA, protein interaction interface (PPI) residence, nearest neighbor count (Nc), and nearest neighbors with known function (KNc)–are strongly predictive for KF-MAPs [2], but suffer from the loss of any one ANN input factor. Therefore, to compensate for the loss of structural features used in our previous model (SASA and PPI) and to determine optimal combinations of input factors for the prediction of function potential, we systematically evaluated all possible combinations of eight protein sequence and familial features including the four previously-evaluated structure-free parameters and four additional untested features. Untested features included the organism ID count (OIDc), mass spectrometry source count (MSSrc), neighbor PTM count (NPTMc), and disorder tendency (DIS) (see methods for a full description of each).

ANN models were created for every possible linear combination of the eight input factors, yielding 247 unique input feature sets. For each feature set combination (e.g. PTMc + MSsrc + PRC) we generated 100 ANN models wherein each model was trained with a unique and randomly determined training and validation set to produce 24,700 individual ANN models with unique input layer and sampling combinations. We then assessed the predictive ability of specific factors from the aggregate statistics across all 100 sample iterations and used ROC-AUC metrics to rank order ANN performance. The ROC curve plots the rate of true positive discovery versus false positive with the point (0,1) being the optimum in which all true positives have been identified without the incorporation of false positives. A ROC-AUC of 1.00 represents a perfect model whereas a model with a ROC-AUC of 0.50 is no better at distinguishing the two classes of positives than would be expected by random assignment. Models with ROC-AUC of 0.75 or higher are generally considered excellent predictors.

As each of the 100 random holdback sets was kept consistent across each set of ANN input factors, we first assessed the variability amongst training datasets across all factor combinations. We observed little variation in aggregate ROC AUC metrics for any of the 247 input layers between randomized training sets. The mean standard deviation for all input layers across all training sets was 0.0016 (S1 Table). This consistency indicates that all 247 ANN input layers are resilient with respect to variability in training data sampling. Furthermore, the standard deviation in ROC AUC across each input factor set was similarly stable—ranging from 0.0424 to 0.0498. At this level of abstraction, any variability or potential under/over-fitness was not sufficient to skew the aggregate statistics.

**Fig 2:**
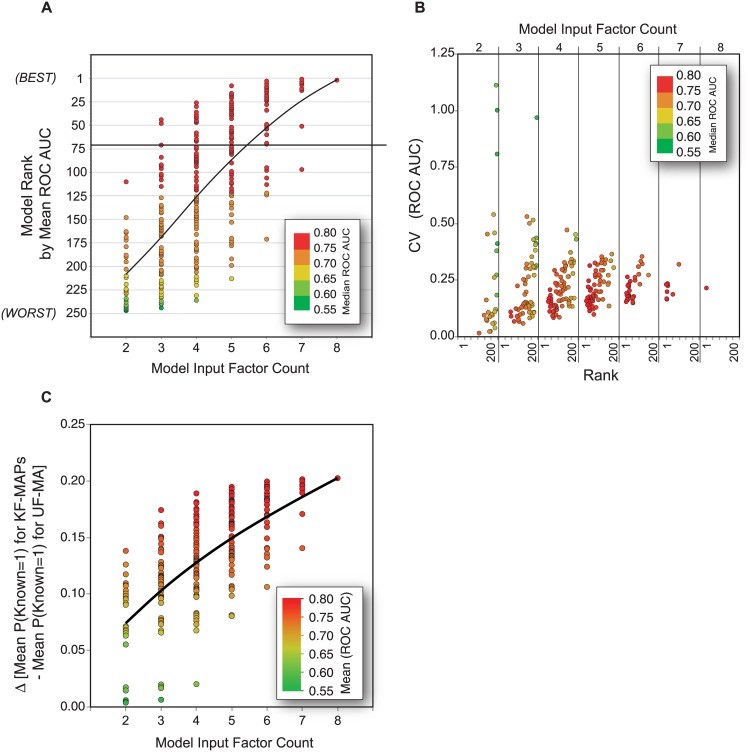
Performance characteristics of variable input layer ANN models trained to predict known-functional MAPs. **(A)** Graphical display of the ranking of ANN model performance as measured by mean ROC AUC (y-axis) and median ROC AUC (color scale) as grouped by the number of factors included in the neural network input layer (2–8; x-axis). The y-axis is reversed such that model ranking proceeds from highest (1) at the top to lowest (247) near the x-axis intercept. **(B)** Coefficient of variation (CV) for 247 ANN models shown in (A). **(C)** Mean difference in probability between KF-MAPs and UF-MAPs, indicating dynamic range of the probability response for each model.

Direct comparison of the mean ROC AUC relative to the model input factor count for each of the 247 models revealed a clear trend showing that an increase in the number of input factors leads to an overall increase in the average mean and median ROC-AUC—indicating that the inclusion of a greater number of inputs generally improves prediction strength (Fig 2A). Higher factor inclusion also resulted in more robust performance between randomized training/validations sets. For example, 2 and 3-factor models exhibited a broad range of coefficient of variation (CV) values, some of which were 100% or higher—likely dependent on a lack of robust behavior in face of an “unfavorable” randomized training/validation dataset (Fig 2B). With an increase in factor count, the CV decreased and stabilized at ~21% for the 8-factor model—likely reflective of the existence of functional PTMs that have yet to be discovered (i.e. proven functional by empirical methods) and are therefore “mis”-classified with UF-MAPs. While some low-factor-count models exhibit very low CV (e.g. KNc-MSSrc CV = 1.6%), they also suffer from compression in the ANN output probability scale within which KF- and UF-MAPs must be distinguished. For example, despite the apparent optimal performance of KNc-MSSrc 2-factor models (ROC AUC = 0.75), the mean difference in ANN probability scales for KF- and UF-MAPs is 1.5-fold lower than the mean difference for the top 7-factor model (Fig 2C). Thus, maximizing robustness and performance of the model is accomplished at the expense of dynamic range. We conclude that structure-free ANN models perform as well if not better than our previously derived structure-dependent ANN model for the ranking of PTM function potential. Furthermore, increasing the number of ANN input factors generally increases model performance and robustness, and several different models that rely on different input factor combinations can be used to achieve a similar degree of performance.

### Interdependence of input factors in high-ranking ANN models lacking MSSrc

**Fig 3:**
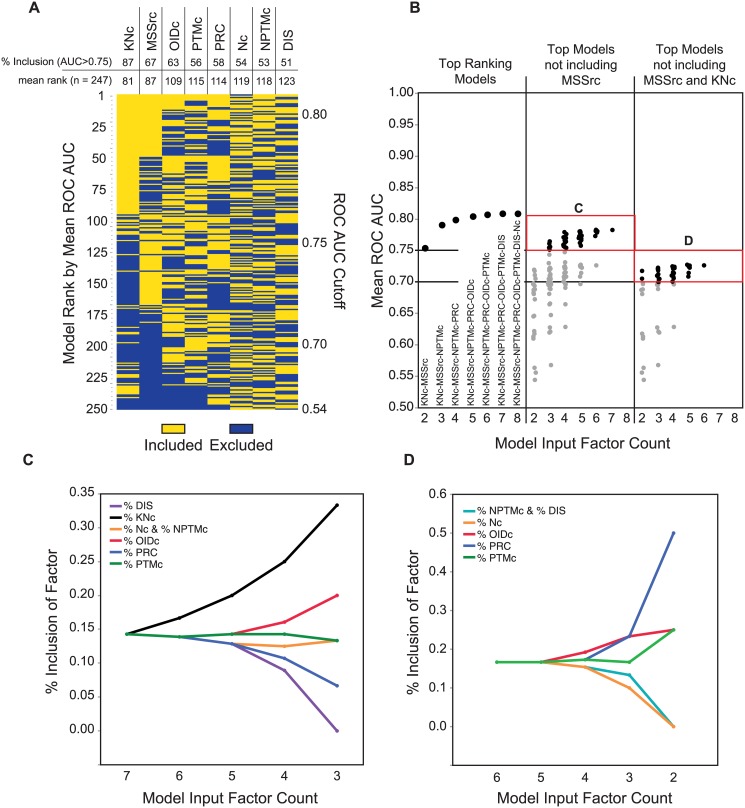
MSSrc and KNc are predominant input factors found in high-ranking ANN models, but are not essential for high performance prediction. **(A)** Rank order hierarchical clustering of ANN models with variable input factor inclusion. Input factors included in each model (yellow boxes) compared with factors not included (blue boxes). Models ranked in same order as in Fig 2A. % inclusion of each factor in models with mean ROC AUC > 0.75 are indicated (top; upper number). The mean rank determined by the average of rank positions for each factor are indicated (top; lower number). **(B)** Comparison of mean ROC AUC for top ranking models at each input layer level (left); for top models lacking MSSrc as an input factor (middle); and for top models lacking both MSSrc and KNc as input factors (right). Red boxes (C and D) correspond to the subset of data used for factor inclusion analysis shown in panels C and D. **(C)** Percentage of inclusion observed for the indicated factors with respect to decreasing model input factor count in cases where MSSrc is not included. **(D)** Same as in C, but for models lacking both MSSrc and KNc. Lines trending upward indicate increase in the inclusion of these factors in small input layer/high performance models shown in the red boxes of Fig 3B.

When ranked by mean ROC AUC, the top-performing model is a 7-factor model including PTMc, NPTMc, KNc, PRC, DIS, MSSrc, and OIDc, which resulted in an average ROC AUC of 0.8085. However, several models were identified wherein the ROC AUC was greater than 0.75 (rank order 120 or higher). Of these top 120 models, MSSrc and KNc were the most predominant features. Indeed, 87% of the top 120 models included KNc and 67% including MSSrc (Fig 3A). This scaled to 100% and 80% above ROC AUC 0.773, which reflects the maximum performance of our previous 6-factor model that included SASA and PPI data derived from 3-dimensional protein structures [2]. We also found that other combinations of input factors were frequently co-incident in higher-performing models and that this relationship changed depending on whether or not KNc or MSSrc were also included (Fig 3A, yellow boxes). Indeed, the top-ranking model at each factor count (2–8) built on these two factors (Fig 3B, left panel).

MSSrc, which is proportional to the frequency of experimental observation of a PTM, is a commonly used feature for prediction of biological importance wherein PTMs with greater MSSrc are considered more likely to be functionally important. This is true in some but not all cases. Furthermore, acquisition of MS data is also extraordinarily labor and cost intensive (from a community perspective), which warrants further investigation as to its value as a predictive feature in models aimed at identifying function potential of PTMs. We found that 25% of all KF-MAPs have been reported only once (MSSrc = 1), indicating that higher MSSrc is not a perfect predictor of function (S2 Table). In contrast to MSSrc, KNc reflects the current state of experimental coverage by the field at large—and is less susceptible to experimental control by any one individual. Like MSSrc, KNc is not itself a perfect predictor of biological function due in part to the observation that 78% of all KF-MAPs have no neighboring MAPs with known function (KNc = 0).

To evaluate the impact of MSSrc on ANN models and the input factors contained therein, we compared the mean ROC AUC for models lacking MSSrc or MSSrc and KNc (Fig 3B, middle & right panels). The highest performing model lacking MSSrc as an input factor is a 6-factor model with a ROC AUC of 0.783; and includes all features except MSSrc and Nc (S1 Table). However, several models are found that lack MSSrc as an input factor but achieve a mean ROC AUC > 0.75 –some of which also have less than 6-factors. Thus, while multiple feature combinations can be found that circumvent the need for MSSrc, not all combinations are capable to do this. Therefore, to determine which factors are most critical for achieving high performance in the absence of MSSrc, we analyzed the percentage of each factor’s inclusion in models above mean ROC AUC 0.75 with respect to input factor count (Fig 3B, middle panel red box). In this case, factors that contribute significantly to small input layer/high performance models are expected to exhibit a high frequency of inclusion.

In high performance models that lack MSSrc (Fig 3B; red box C), we found distinctive patterns in the inclusion of specific input factors of small input layer ANN models (Fig 3C). For models that lack MSSrc, KNc and OIDc become dominant features with decreasing factor count, whereas PRC and DIS features are included far less for these high-performance models (Fig 3C). Thus, in the absence of MSSrc, the count of neighboring MAPs with known function and the diversity of the MAP (in terms of the number of organisms represented) become dominant predictive features.

We next evaluated models lacking both MSSrc and KNc—the two most included features found in all high-performance models. The highest performing model lacking MSSrc and KNc as input factors is a 5-factor model with ROC AUC of 0.727 that lacks NPTMc, MSSrc and KNc (S1 Table). Factor inclusion analysis of the highest performing models lacking MSSrc and KNc (ROC AUC > 0.7) (Fig 3B red box D), revealed that OIDc, PRC, and PTMc are dominant features that become enriched in small input layer/high performance models.

**Fig 4:**
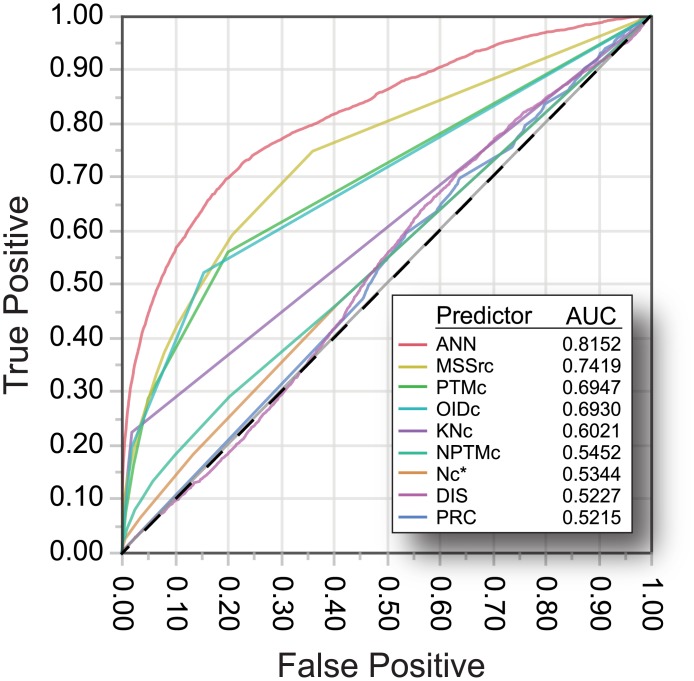
The top-ranked ANN model out-performs the use of single-feature logistic regression models for the prediction of KF-MAPs. ROC curve comparison for the top-ranking ANN model versus single-feature logistic regression models. AUC values shown for the best resulting model (i.e. not the mean AUC for 100 randomized datasets). *The ROC curve for the logistic regression of Nc is included for completeness only and is not included in the input layer for this ANN.

We also evaluated each input factor as a single predictor in logistic regression models. As expected, the top 7-factor ANN model out-performs the logistic regression for any individual input factor in terms of predictive ability (Fig 4). Amongst the eight individual features, MSSrc provides the best overall performance with a ROC AUC of 0.742 (Fig 4). PTMc also provides reasonable performance as a single feature predictor, as we have shown previously [1,2]. Likewise, OIDc, which we have not evaluated previously, is also a relatively strong single feature predictor of functional MAPs (Fig 4). Interestingly, we found that the predictive strength of any single feature used in logistic regression models did not coincide precisely with the enrichment of the feature in ANN models (compare Figs 4, 3C and 3D), indicating that the combination of input factors is responsible for defining predictive strength in the ANN models as opposed to an additive benefit from clustering several moderately predictive features. For example, PRC, which is the least effective single feature predictor, is a dominant factor in low factor-count/high performance ANN models. Similarly, KNc, which is the most predominant ANN input factors in high-performance models, is by itself a weak predictor of known function.

In conclusion, while MSSrc is highly predictive for PTM function (as measured by segregation of KF-MAPs), it is not essential for high performance multi-factor ANN models. Indeed, combinations of moderate single-feature predictors achieve increased predictive power through integration into neural networks. This can be observed through analysis of high performance models that lack MSSrc as an input. We find that lower factor-count/high performance models that lack MSSrc benefit significantly from inclusion of KNc and OIDc that are derived from multiple sequence alignments of protein families. In contrast, models lacking both MSSrc, KNc, while exhibiting relatively diminished performance, are compensated for by combinations of different input factors whose interactions in the neural network provide predictive power. The dominant features in these cases provide a predictive benefit through a combination with other features that is not directly related to the predictive power of any single feature alone.

### Evaluating confidence for the top ANN model and resulting hotspot predictions

**Fig 5:**
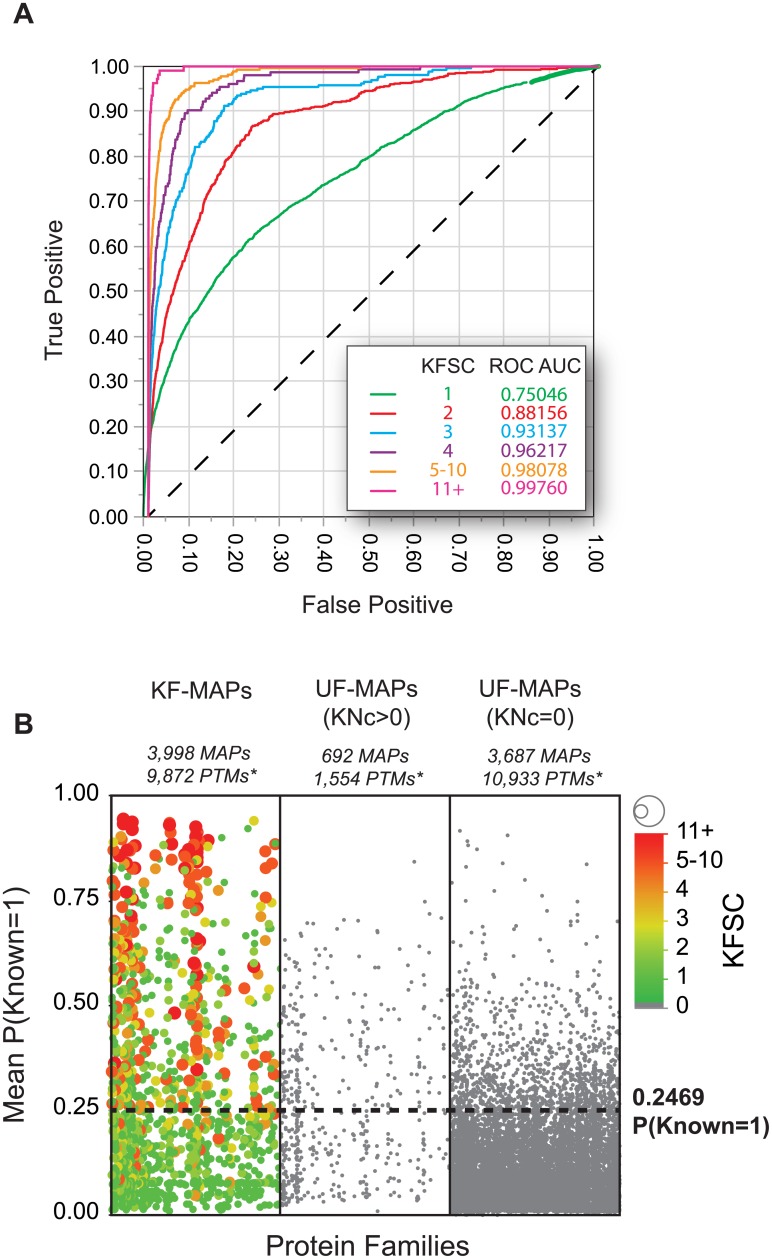
The top-ranking ANN model is a near-perfect predictor of KF-MAPs with 11 or more published sources of experimental validation and identifies ~14,000 PTMs with high potential for biological function. **(A)** Multiple ROC curves—plotted using various KFSC value cutoffs as indicated in the figure legend—for the top-ranking ANN model are displayed. The increasing ROC AUC with increased KFSC indicates the ANN is predictive of known function source counts, which serve as a measure of degree of validation of function and have been excluded from all ANN input layers. **(B)** MAPs belonging to 3 classes are plotted by the predicted probability of known function [P(Known = 1); y-axis]. For clarity in visualization, only a random sample of 1/3 of the 83,438 MAPs are displayed. The left panel displays KF-MAPs where points are additionally scaled in color and size by KFSC. The middle panel displays UF-MAPs with a neighboring (+/-2) KF-MAP (KNc>0). The right panel shows the UF-MAPS lacking neighboring KF-MAPs (KNc = 0), which are more commonly observed throughout the data. A dashed line is shown at P(Known = 1) = 0.2469 –the probability above which 90% of KF-MAPs with KFSC > = 4 are found. This cutoff represents a conservative threshold for segregating UF-MAPs with a high probability of known function. (*MAP and PTM counts are representative of the P(Known = 1) threshold of 0.2469 applied to the entire MAP set—not the 1/3 randomized sample displayed).

The known function source count (KFSC) provides a measure of validation for the identification of biologically functional PTMs—the more often a functional role has been observed, the more confident we can be in the assignment of a functional impact to the PTM [2]. The number of PubMed IDs (PMIDs) associated with an assignment of biological response to a particular PTM (curated in PhosphoSitePlus) was not included as an input factor in any of the ANNs evaluated here. However, KFSC does provide a metric for the distinction of highly verified function from novel functional assignments. A responsive model with sufficient resolution should be able to scale P(Known = 1) values for KF-MAPs with increasing KFSC. To examine this relationship, we plotted the P(Known = 1) values for KF-MAPs as predicted by the top-performing model at various KFSC cutoffs (Fig 5A). Consistent with previous ANN model results, we observed a steady increase in ROC AUC as it approaches peak value with increasing KFSC—indicating that the ANN model is predictive of the degree of validation of biological function. The ROC AUC for the ANN ranges from 0.751 when only known function MAPs with KFSC of 1 are included to 0.9976 when only those with 11 or more sources are included (Fig 5B). Taken together, this data indicates the ANN model is both predictive of the degree of verification for biologically functional MAPs and operates as a near-optimal classifier for those MAPs which contain a large body of evidence for PTM functional impact.

As the ANN model provides increasing predictive strength for KF-MAPs with increasing KFSCs, so should it appropriately rank UF-MAPs that share optimal combinations of input feature values with those of increasing KFSC. Indeed, we find that the ANN model ranked thousands of UF-MAPs as having a high likelihood of functional impact by this criterion. As previously described, we considered UF-MAPs to be *functional-potential hotspots* when P(Known = 1) was greater than or equal to the probability equal to the 90^th^ percentile taken from the distribution for KF-MAPs with KFSC > 4+, which was 0.2469 for this model (Fig 5B) [2]. As such, we classified 4,379 UF-MAPs as function potential hotspots (corresponding to 12,487 PTMs). Of these, 692 MAPs (1,554 PTMs) were classified as being known by proximity (i.e. KNc > 0). Consequently, 3,687 UF-MAPs (10,933 PTMs) were identified as potentially novel regulatory elements—having high function potential without being assumed functional by proximity. This body of modification data represents potential novel PTM regulatory elements and should be considered a priority for investigation into biological function.

## Conclusions

Given the exponential rate of identification of PTMs and the relatively limited availability of refined protein structural data, computational methods for prioritizing modifications on functional bases are critical in moving the field forward. The 2016 release of the dbPTM, which aggregates PTM data from numerous sources, contains ~577,000 experimentally observed, eukaryotic modifications across 59,410 unique proteins—a more than two-fold increase in the number of modifications and proteins included as compared to the 2013 release [27]. During the same period, the number of protein structures deposited in the PDB has grown by less than 30%, and the number of eukaryotic proteins represented has increased by fewer than 9,000 sequences. This increasing data gap must further be addressed by computational methods that employ predictive features not directly linked to structure. To that end, we have demonstrated the predictive power of artificial neural networks models for PTMs in the absence of structural feature data—significantly expanding the body of modification data analyzable for functional prioritization.

We have thoroughly explored the predictive performance of 8 different input features as well as the variability among these features for KF-MAPs. In generating ANNs for each of 247 possible input layers, we have observed a trend towards enhanced robustness with larger input layers. By randomizing holdback data, we have also shown that ANNs generated for this dataset are largely resilient to variability in feature profiles in the training set. Finally, we have generated several highly predictive neural networks for prioritizing PTMs in the absence of structural data.

One of the primary goals of this experiment was to address potential for sampling-dependent variability among ANN models of various input layer size and composition. Each of the 24,700 individual ANN models represents a set of scalar weights applied uniquely by each node (perceptron) in the network in conjunction with the parameterized activation function. The training of each model is accomplished by dividing the whole dataset into separate training and validation (or testing) subsets—the size of which are determined by the holdback parameter. If biologically functional MAPs are heterogeneous with respect to their input factor values, a “poorly” selected training set could lead to a bias in the ANN which systematically diminishes the probabilities in another subset of functional MAPs within the validation dataset. In order to assess the potential for biased holdback, we applied each input factor set to 100 randomly partitioned training and validation datasets. Examination of the aggregate statistics for ROC AUC and the distribution of P(Known = 1) for KF-MAPs versus UF-MAPs were expected to highlight these potential mis-fit models.

### Assessing ANN performance variability

As the features that define biologically functional PTMs are not fully understood, we are currently unable to develop models that directly classify PTMs based on functional consequence. However, utilizing the body of experimental evidence, we are able to quantify numerous features of modified residues that exhibit biological function. Neural networks trained to identify these KF-MAPs weigh their associated input features and rank the remaining UF-MAP data accordingly by the degree to which they share these features. While there are several other machine learning classifiers that may be utilized instead of single layer ANN models (e.g. Bayesnet, Logistic, Random Forest, Multi-Layer perceptron, support vector machines), existing evidence does not suggest that they differ considerably in performance for functional prioritization of PTMs [28]. In contrast, we show here that input factor set has a pronounced impact on functional prioritization. By evaluating all possible input factor set combinations across 8 established features, we highlight multiple “productive” sets and produce a model that conservatively identifies ~11,000 distinct PTMs with high predicted probabilities of known function which may represent novel regulatory functions and should be considered priority research targets.

An interesting outcome of these experiments is lack of variability with respect to the mean ROC AUC metric amongst many of the different ANN models. The top 25 models, for example, vary in mean ROC AUC less than 1% in their cumulative distribution with the top-ranking model at 0.8085 and the 25^th^ ranking model at 0.7987. Furthermore, a model is considered a good predictor when the ROC AUC is greater than 0.75, which extends the range to the 120^th^ ranked model and includes ANNs of all factor counts. When we examine the mean rank position based on the inclusion of each individual factor, we observe two outliers: one, MSSrc whose mean model rank by inclusion is 86.68; and two, KNc with the highest mean rank of 80.76. The remaining factors are more narrowly distributed with OIDc highest with 108.64 mean rank, and DIS as the lowest at 123.08. Of particular note is the high-ranking outlier in the 2-factor column, which represents the ANN derived from the KNc-MSSrc input layer. This model is ranked 114^th^ and with a ROC AUC of 0.7537 is considered a good predictor of MAPs containing PTMs of known biological function. In some such cases, sufficiently discriminatory class-decision ANNs can be developed from relatively small input layers.

While several potential ANN models adequately separate populations of KF-MAPs from UF-MAPs in probability space, the true positives (KF-MAPs) and their respective P(Known = 1) values serve as internal validation controls. A theoretical “perfect” (or overfit) ANN model would yield a P(Known = 1) of 1.0 for all KF-MAPs and 0.0 for all UF-MAPs. With such an extreme distribution of probabilities, the output would be of no use for the prioritization of function studies into PTMs contained within UF-MAPs—which comprise more than 95% of MAP datapoints. Indeed, the value for the research community lies in the ability of the ANN to distribute MAPs along the 0.0–1.0 continuum so that, by comparison with KF-MAP metrics, enable distinction of high from low probability function potential. We have shown that small feature layer sets can lead to compression in the range of predicted probabilities—producing less favorable outcomes compared to those generated by larger feature set models. However, this depends greatly on the choice of factors that are included.

### Expanding the input factor set

Although we have assessed the predictive performance of a wide array of protein and PTM features in this study, the included set of inputs is not exhaustive. Additional features from the field of proteomics as well as genomics could be included in future models and may prove predictive of PTM function. We have currently restricted our analysis to experimentally verified data from such fields, since it grounds the predictive model on realized, as opposed to predicted, PTM site data. However, tools that have been used successfully in identifying predictive features in proteins, such as those based on combinations of amino acid sequence order, may also provide yet additional features and improvements that may enhance PTM function potential prediction [29].

Any model is expected to improve as more biologically responsive PTMs are identified and reported in the literature—thereby increasing the size of training data. Indeed, the primary limitation for this computational approach is the availability of data. The segment of the proteomics field dedicated to the study of PTMs lacks a centralized repository for data concerning both the observation of modifications via mass spectrometry as well as experimental confirmation, or lack thereof, of biological function. The existing, fragmented network of public repositories—mostly modification type- or organism-specific—have widely variable methods for reporting and accessing data. This presents significant challenges to computational approaches to the study of PTMs. A common platform for deposition and curation of modification-related data, akin to the PDB for 3D structure, could greatly improve the pace of discovery.

## Supporting information

S1 Fig A schematic representation of the generalized method for analysis. PTM data from the 2013 release of dbPTM undergoes manual curation prior to inclusion in the data. Canonical protein sequences and familial classifications are obtained from UniProt and InterPro, respectively. Data identifying PTMs with known biological function is obtained from PhosphoSitePlus (PSP). The SAPH-ire core is used to organize these input features and generate additional inputs both via internal (e.g., calculation of conservation of modifiable residues) and external (e.g., IUPred) methods. The resultant MAP data is randomly divided into 100 training/validation sets. The neuralnet package within R generated neural network models trained to predict the probability that a MAP is associated with a known function (PSP data). 24,700 neural networks were created by the combination of 247 possible input layers and 100 sample training sets. The result contains more than 2 billion predicted MAP probabilities of known function. Additional data from PSP indicating the number of independent verifications of biological function (via unique PubMed IDs) bypasses the input layers and is used to further assess the quality of the output models.
(PDF)

S1 Table Aggregate statistics for 247 different input factor layer ANN models. The aggregate statistics for each of 247 possible input factor layers used in the construction of 100 ANN models based on 100 different holdback-randomized datasets. Each dataset is comprised on 83,438 MAPs, 3,998 of which are classified as functional (Known = 1). (Sheet 1) Column legend. (Sheet 2) Table.
(XLSX)

S2 Table ANN-derived function potential probabilities (P(Known = 1)) for the top performing input factor layer model. All factors except for Nc were included for model training. (Sheet 1) Column legend. (Sheet 2) Table with factor data and mean, standard deviation, minimum, maximum, and median values shown.
(XLSX)
