# Supplementary figures and images for: Systematic analysis of non-structural protein features for the prediction of PTM function potential by artificial neural networks
Source: PLoS One. 2017 Feb 22;12(2):e0172572. doi: 10.1371/journal.pone.0172572 (PMC5321281; doi:10.1371/journal.pone.0172572)

Fig. S1

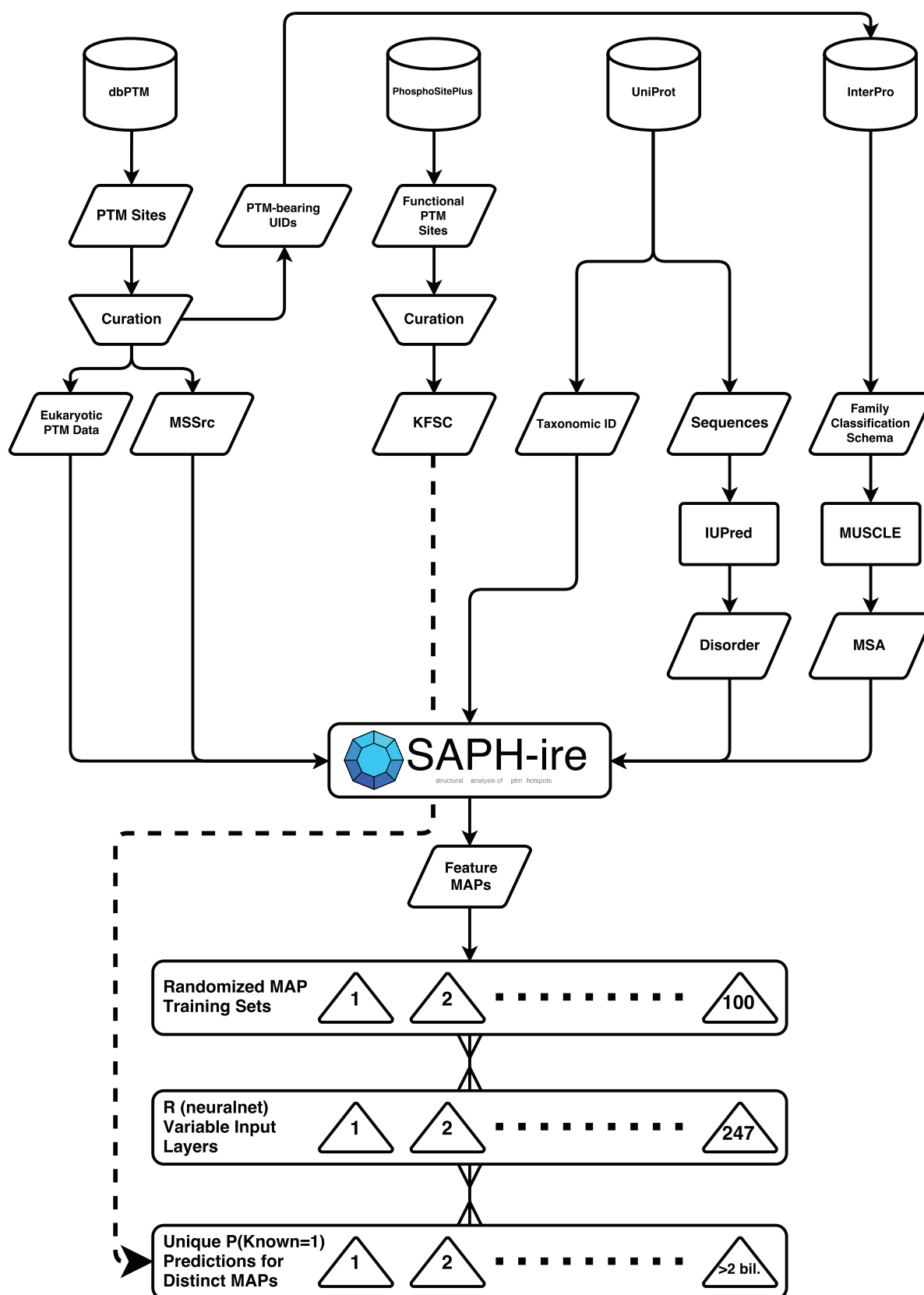

Supplement: S1 Fig — PTM data from the 2013 release of dbPTM undergoes manual curation prior to inclusion in the data. Canonical protein sequences and familial classifications are obtained from UniProt and InterPro, respectively. Data identifying PTMs with known biological function is obtained from PhosphoSitePlus (PSP). The SAPH-ire core is used to organize these input features and generate additional inputs both via internal (e.g., calculation of conservation of modifiable residues) and external (e.g., IUPred) methods. The resultant MAP data is randomly divided into 100 training/validation sets. The neuralnet package within R generated neural network models trained to predict the probability that a MAP is associated with a known function (PSP data). 24,700 neural networks were created by the combination of 247 possible input layers and 100 sample training sets. The result contains more than 2 billion predicted MAP probabilities of known function. Additional data from PSP indicating the number of independent verifications of biological function (via unique PubMed IDs) bypasses the input layers and is used to further assess the quality of the output models. (PDF) [file pone.0172572.s001.pdf]
